# The Mediating Role of Screen-Based Sedentary Behaviors in the Association of Parental Educational Level and BMI with Preschoolers’ Ultra-Processed Food Consumption

**DOI:** 10.3390/nu18071069

**Published:** 2026-03-27

**Authors:** Aristides M. Machado-Rodrigues, Helder Miguel Fernandes, António Stabelini Neto, Elizabete Alexandre Dos Santos, Josep A. Tur, Cristina Padez, Daniela Rodrigues

**Affiliations:** 1 University of Coimbra, Faculty of Sport Sciences and Physical Education, 3040-248 Coimbra, Portugal; 2 Interdisciplinary Centre for the Study of Human Performance (CIPER), University of Coimbra, 3040-248 Coimbra, Portugal; 3 Research Centre for Anthropology and Health, University of Coimbra, 3001-401 Coimbra, Portugal; elizabete.nutri21@gmail.com (E.A.D.S.); cpadez@antrop.uc.pt (C.P.); rodrigues1323@gmail.com (D.R.); 4 School of Education, Communication and Sports, Polytechnic Institute of Guarda, 6300-559 Guarda, Portugal; hmfernandes@ipg.pt; 5 Sport Physical Activity and Health Research & Innovation Center (SPRINT), 6300-559 Guarda, Portugal; 6 Health Sciences Center, State University of Northern Paraná, Jacarezinho 86400-000, Brazil; asneto@uenp.edu.br; 7 Department of Nutrition, Paulista School of Medicine (EPM) of the Federal University of São Paulo (Unifesp), Botucatu Street, 720, Vila Clementino, São Paulo 04023-062, Brazil; 8 Research Group on Community Nutrition & Oxidative Stress, University of the Balearic Islands—IUNICS, 07122 Palma de Mallorca, Spain; pep.tur@uib.es; 9 CIBEROBN (Physiopathology of Obesity and Nutrition)-Instituto de Salud Carlos III, 28029 Madrid, Spain; 10 Health Research Institute of Balearic Islands (IdISBa), 07120 Palma de Mallorca, Spain

**Keywords:** ultra-processed foods, screen time, preschool children, parental BMI, mediation analysis

## Abstract

**Background/Objectives**: The mediating role of the diverse range of screen-based sedentary behaviors (SBs) remains understudied, particularly at younger ages. The present study examined the direct and indirect effects of parental BMI and education on ultra-processed food (UPF) consumption among preschoolers, testing the potential mediating role of screen time. **Methods**: The cross-sectional study sample comprised 919 kindergarten children (484 boys, 52.7%), with ages ranging from 2.2 to 6.8 years (mean: 4.7 ± 1.0 years). Screen-based sedentary behaviors (television viewing, smartphone use, tablet use, computer use, and playing electronic games) were measured by proxy-report fulfilled by parents, separately for weekdays and weekends. UPF consumption (drinks/yogurts, packaged/fast foods, and sweet/salty snacks) was assessed via 24 h recall scales. Path analysis mediation models tested direct effects of maternal/paternal BMI and education on UPF intake, and indirect effects through screen time, controlling for child age and sex. **Results**: Lower parental education and higher parental BMI were associated with increased mobile device use and UPF consumption (r = 0.10–0.28). Screen-based sedentary behaviors mediated the association between maternal BMI and UPF pathways (15–90% of total effects), particularly for sweet and salty snacks (50–90%). Parental education effects were also mediated by screen time (9–23% indirect effects), with paternal education showing stronger protection against packaged/fast foods. **Conclusions**: Mobile devices and watching television partially mediate intergenerational transmission of obesogenic dietary patterns from parental BMI/education to preschoolers’ UPF consumption. Findings of the current study support family-centered interventions targeting screen-time limits and UPF exposure, mainly at the weekends, to prevent early obesity trajectories.

## 1. Introduction

Early childhood constitutes a sensitive period for the development of long-term behaviors related to diet, habitual physical activity, and overall lifestyle. During these initial developmental years, the family context plays a crucial role in shaping children’s behavioral patterns, with parents serving as key models and regulators of daily routines. Some biological and social parental features, such as body mass index (BMI) and educational level, have been widely recognized as important determinants of children’s health-related practices, including their physical activity and dietary behaviors [1,2]. These factors influence the home food environment, parenting practices, and the overall structure of children’s daily routines, thereby establishing early pathways toward either protective or obesogenic trajectories [3,4]. Evidence from Portuguese cohorts, such as the Generation XXI study, has shown that parental BMI and sociodemographic characteristics strongly predict children’s weight status and lifestyle behaviors during the preschool years [5,6].

Parental educational attainment and nutritional literacy shape home food environments and children’s subsequent preferences. Families with higher education levels are more likely to implement healthy eating practices and restrict access to energy-dense snacks and ultra-processed food (UPF) [6,7]. Conversely, parents with limited educational resources may face multiple barriers, including financial constraints, time scarcity, or limited nutrition knowledge, which can increase dependence on readily available UPF [8]. In Portugal, epidemiological studies have documented a socioeconomic gradient in UPF consumption among children, with lower parental education associated with higher intake of processed snacks, sugary beverages, and ready-to-eat meals [6,9]. Globally, recent data have also indicated that UPF contributes a substantial and growing proportion of total energy intake in childhood, suggesting that in some pediatric populations, nearly half or more of daily energy intake derives from UPF [10]. The overconsumption of these products, typified by excessive energy density, added sugars, saturated fats, and sodium, and poor micronutrient content, has been consistently linked to metabolic disturbances, excess adiposity, and early-onset obesity and related cardiometabolic risk among children [11].

UPF is frequently associated with sedentary behaviors, particularly those involving prolonged exposure to screen-based activities [12]. Among young children, increased television viewing, tablet use, and smartphone interaction have been associated with higher energy intake, reduced physical activity, elevated exposure to persuasive food marketing promoting UPFs [13], and cardiometabolic risk [11]. In Portugal, national surveys have reported that screen time among preschool-aged children often exceeds health recommendations and correlates with elevated consumption of sugary and ultra-processed foods [5,14]. In addition, recent epidemiological studies with school-aged children and their parents further indicate that less healthy parental lifestyles—including higher screen time and UPF intake—are closely associated with children’s own UPF consumption and sedentary behaviors, reinforcing the pivotal role of familial patterns in shaping obesogenic profiles [13,15]. Beyond displacing active play, screen-based behaviors are thought to reinforce inattentive eating and impair satiety cues, thereby potentially mediating the association between parental characteristics and children’s dietary intake [13].

On the other hand, further recent international research suggests that the type of sedentary behavior exerts a differential impact on dietary patterns [16]. Evidence from large-scale studies across Europe, North America, and Latin America shows that passive screen time, particularly television viewing, is more strongly associated with unhealthy food consumption and increased BMI than interactive or educational uses of digital devices [6,10,13]. In Portugal, previous studies have also highlighted that the TV viewing exposure is most consistently associated with unhealthy snacking and UPF intake, while the use of tablets or computers for educational purposes shows weaker or contradictory associations [13,17]. Moreover, previous complementary studies have also emphasized that high screen time tends to cluster with several unhealthy behaviors, not just with frequent UPF consumption, but also irregular meal patterns, as well as insufficient daily physical activity, across childhood and with a high likelihood to follow the individuals to adolescence [5,11]. This growing, challenging, and somewhat inconsistent evidence underscores the importance of examining the mediating role of specific, diverse screen-based sedentary behaviors in the intergenerational transmission of dietary habits and obesity risk.

**Figure 1:**
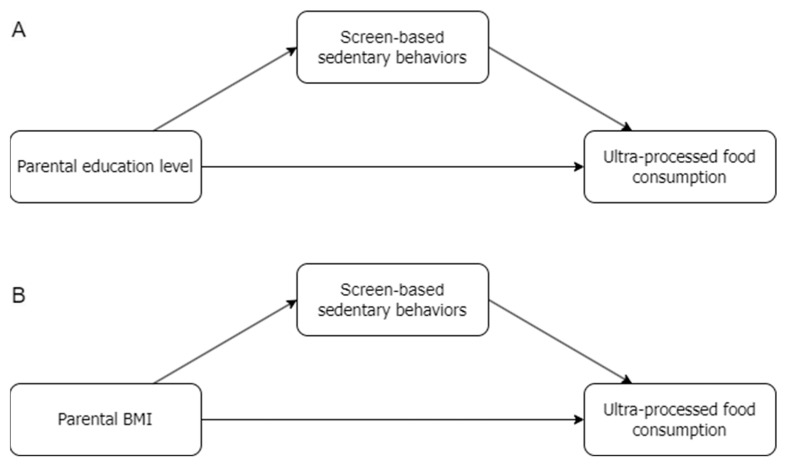
The hypothesized mediation models. (**A**) The mediation model for the effect of screen-based sedentary behaviors on the relationships between parental education level and children’s ultra-processed food consumption; (**B**) the mediation model for the effect of screen-based sedentary behaviors on the relationships between parental BMI and children’s ultra-processed food consumption.

In the context of the preceding trends, especially in preschoolers who are more dependent on parents, further research is needed to contribute to potential prevention strategies against sedentary behaviors and pediatric obesity, which should incorporate both family rules and child behavior-centered dimensions. Therefore, the current study aims to evaluate the specific effects of parental BMI or educational level on UPF consumption among kindergarten-aged children, examining the mediating role of screen-based sedentary behaviors. It was hypothesized that screen-based sedentary behaviors would mediate the association between parental BMI or education levels and the child’s consumption of ultra-processed foods in Portuguese preschoolers. Parental influences were examined in separate models because they capture different, albeit interrelated, dimensions of the home environment that may shape preschool children’s ultra-processed food consumption through partially distinct pathways, namely familial weight-related behavioral patterns in the case of BMI, and socioeconomic and health-literacy resources in the case of educational attainment. The hypothesized mediation models are shown in Figure 1.

## 2. Materials and Methods

### 2.1. Participants and Study Design

The current cross-sectional study is part of the *ScreenHealth Project* (https://screenhealth.uc.pt/), which is the first longitudinal study of Portuguese preschoolers aiming to investigate screen media use trajectories and their interactions with 24 h behavioral movements (e.g., active and sedentary behaviors, and sleep duration) as well as dietary patterns, and their subsequent impact on pediatric obesity. In 2024, parents of children attending 132 daycare centers and preschools in Coimbra were invited to participate in the project. The first wave (April–June 2024) included 1050 participants drawn from 60 kindergarten classrooms in both public and private schools. Participants who did not respond to the invitation or refused to participate were excluded. The current sample is composed of 919 preschoolers, including 484 boys (52.7%) and 435 girls (47.3%), with ages ranging from 2.2 to 6.8 years (mean: 4.7 ± 1.0 years). The children’s BMI z-scores varied from −2.1 to 5.4 (0.5 ± 1.0). The average age of mothers was 38.1 ± 5.5 years, with most of them (60.4%) having completed sixteen or more years of education. Maternal BMI ranged from 15.4 to 42.4 kg/m^2^, with 33.6% classified as overweight. The average age of fathers was 40.0 ± 5. 9 years, and 43.4% also had sixteen or more years of formal education. The father’s BMI ranged from 16.9 to 42.0, with 51.9% classified as overweight.

All participants provided written informed consent. The study protocol was approved by the Ethics Committee of the Interdisciplinary Research Institute of the University of Coimbra (CEIIIUC, REF 1_ID312; 26 July 2023) and was conducted under the supervision of the University’s Data Protection Officer.

All variables were collected using questionnaires completed by parents at home. Parents were asked to return the completed questionnaires to the schools in sealed envelopes to ensure data confidentiality. Children whose parents did not respond to the invitation, refused to participate, or did not return the questionnaire were excluded from the initial sample recruitment.

### 2.2. Anthropometry

Initially, parents reported their children’s and their own sociodemographic characteristics (age and sex). Children’s height and weight were objectively measured using a calibrated SECA (Birmingham, UK) portable stadiometer and a digital scale (SECA, United Kingdom). Children’s anthropometric indices were assessed by trained researchers, using standardized techniques and calibrated equipment; height was recorded to the nearest 0.1 cm and weight to the nearest 0.1 kg. Children’s BMI (kg/m^2^) was computed and converted to age and gender specific z-scores by using the WHO growth charts. In contrast, parents self-reported weight in kilograms and height in meters. Those data were then used to compute the mothers’ and fathers’ BMI.

### 2.3. Ultra-Processed Food Consumption

The NOVA-UPF screener [18] was used to assess the consumption of UPFs among youth [12,19], using a no/yes response option (scores of 0 or 1, respectively). Parents reported on their children’s consumption of UPFs over the past 24 h, outside the home, and during screen time. The NOVA-UPF questionnaire includes a list of 27 potential drinks/foods, organized into three main categories: drinks and yogurts (DYs; consists of six items and has a score range of 0 to 6 points), packaged and fast food (PFF; comprises eleven items with a score range of 0 to 11 points), and sweet and salty snacks (SSSs; includes ten items and score range of 0 to 10 points). This structure allows for the calculation of specific category scores, as well as a total UPF score, which ranges between 0 and 27 points [20,21]. This tool is simple and quick to complete and imposes a low respondent burden, making it feasible for the rapid quantification of UPF consumption.

### 2.4. Screen Time

Parents were asked to complete a questionnaire that gathered information on their children’s screen time habits. The assessment of screen-based sedentary behaviors included the average daily duration, measured in minutes, that children typically spend on various devices, such as television viewing, playing electronic games, computer use, tablet use, and smartphone use, during both weekdays and weekends.

### 2.5. Parental Education

Educational background of fathers and mothers was used as a proxy for socio-economic status. Parental educational levels were collected via self-report, asking parents/tutors about their highest level of completed schooling, which was then converted into years of education. Similar procedures have been used in the Portuguese context [18,19].

### 2.6. Statistical Analyses

The initial inspection of the data was conducted to ensure accuracy and identify any outliers. Descriptive statistics, including mean, standard deviation, minimum, and maximum, were used for data analysis, while zero-order Pearson correlations were employed to explore the relationships between variables. A statistical significance level of 5% was established.

The mediation analysis was conducted using the mediation analysis module in JASP, which is based on the lavaan package [22] and allows simultaneous testing of multiple mediators. Missing data were handled using the full-information maximum-likelihood approach. The significance of total, direct, and indirect effects was assessed through bootstrapping, with 95% confidence intervals calculated using the bias-corrected percentile method (2000 resamples, Maximum Likelihood estimator). Effects were considered significant when the confidence intervals did not include zero.

All analyses were completed using JASP software, version 0.95.4 [23].

## 3. Results

Table 1 presents the levels of screen time-based sedentary behaviors and the consumption of UPF among children, along with their zero-order correlations with parents’ BMI and educational level.

Results show that, according to parents, watching television is the most frequent screen-related sedentary behavior among preschool children, both on weekdays and weekends. The use of smartphones and tablets was the second- and third-most common activities, respectively, whereas the use of computers and gaming consoles was minimal. Compared to weekdays, screen time on weekends is significantly higher, nearly doubling for smartphones and increasing nearly sevenfold for gaming consoles. Furthermore, entertainment was the predominant content category (58.5%), followed by educational content (15.9%) and communication (5.1%). Concerning UPF consumption, results indicate a small to moderate intake of various ultra-processed foods (UPFs) and drinks, with minimal consumption during screen time and/or outside the home, as reported by parents.

Additionally, screen-based sedentary behaviors and UPF consumption showed consistent, albeit small-to-moderate, associations with parental educational levels and anthropometric characteristics. Both parents’ educational attainment was inversely related to the children’s intake of UPFs and drinks (and the total UPF score), as well as to the specific use of mobile devices, particularly smartphones and tablets. In addition, positive correlations were observed between both fathers’ and mothers’ BMI and the increased use of mobile devices (smartphones and tablets), along with the consumption of UPF drinks and yogurts. Children’s consumption of packaged and fast UPFs, as well as the total score, was positively associated only with the mother’s BMI.

Preliminary correlation analyses indicated that parental education levels were inversely associated with parental BMI in both mothers (*r* = −0.18, *p* < 0.01) and fathers (*r* = −0.15, *p* < 0.01), suggesting that lower educational attainment was modestly related to higher BMI. Conversely, maternal and paternal BMI were positively correlated (*r* = 0.26, *p* < 0.01), as were maternal and paternal educational levels (*r* = 0.49, *p* < 0.001). Accordingly, maternal and paternal BMI and maternal and paternal educational level were specified as correlated exogenous variables in separate mediation models.

Table 2 and Table 3 display the results of the direct effects analysis of parents’ BMI on the consumption of UPF among kindergarten children, as well as the indirect effects of screen-based sedentary behaviors, separately for weekdays and weekends. For clarity purposes, only the estimates for the previous 24 h UPF scales’ scores are reported.

The results presented in Table 2 and Table 3 indicate that screen-related sedentary behaviors during both weekdays and weekends act as a mediator in all the relationships between maternal BMI and children’s intake of UPFs. On weekdays, the significant total indirect effects accounted for 21% (drinks and yogurts), 15% (packaged and fast foods), and 50% (sweet and salty snacks) of the total effects. On weekends, the indirect effects accounted for 24% (drinks and yogurts), 19% (packaged and fast foods), and 90% (sweet and salty snacks) of the total effects. Specific mediation effects were observed for the time children spent watching television and using tablets or smartphones, with 95% confidence intervals ranging from 0.001 to 0.035. These findings suggest that higher maternal BMI is associated with increased consumption of certain ultra-processed foods and drinks, whereas a considerable portion of this association can be attributed to the greater time children spend in screen-related sedentary activities during the week. Together, parents’ BMI and children’s sedentary behaviors explained between 4% and 9% of the variance in the scores for the UPF scales.

Table 4 and Table 5 show the results of the analysis on the direct effects of parents’ educational level on the consumption of UPF among preschool children, as well as the indirect effects of screen-based sedentary behaviors, differentiated by weekdays and weekends. For clarity purposes, only the estimates for the previous 24 h scores of UPF scales are reported.

Results presented in Table 4 and Table 5 show that screen-related sedentary behaviors during both weekdays and weekends serve as a mediator in all the relationships between maternal and paternal education level and children’s consumption of UPFs, except for the maternal education effect on the intake of packaged and fast foods during the weekdays. The significant total indirect effects represent a small to moderate portion of the total effects, ranging from 9% to 23%. Significant mediation effects were observed for the time children spent on tablets or smartphones, with 95% confidence intervals ranging from −0.057 to −0.001. These findings show that parents with higher educational levels are more likely to have children who engage in less screen-based sedentary behavior, which, in turn, leads these children to consume fewer ultra-processed drinks and foods. Moreover, the results suggest that fathers’ educational level has a stronger protective effect on children’s intake of packaged and fast foods, as well as sweet and salty snacks, on both weekdays and weekends. Collectively, parental educational levels and children’s sedentary behaviors explained between 4% and 14% of the variance in the scores for the UPF scales.

## 4. Discussion

The present study contributes to the growing body of evidence on early determinants of obesogenic dietary patterns by demonstrating that parental BMI and educational levels are associated with preschoolers’ UPF consumption, both directly and indirectly through screen-based sedentary behaviors. Findings of the current study revealed that children whose parents showed higher BMI and lower educational attainment were more likely to engage in higher screen time as well as to consume higher levels of UPF, including drinks and yogurts, packaged and fast foods, and sweet and salty snacks, with small-to-moderate associations (r = 0.10–0.28). These results are consistent with recent studies of Brazilian [24], Finnish [25], Polish [26], Belgian, Bulgarian, German, Greek, and Spanish [27] preschoolers. Equally relevant is its close association with key health markers, underscoring the urgency of potential early life interventions. Longitudinal and clinical evidence [6,11,28] indicates that early and sustained exposure to ultra-processed foods (UPFs) is linked to adverse cardiometabolic risk profiles and trajectories of increasing adiposity throughout childhood. These findings further emphasize the critical role of family-level socioeconomic and weight-related factors in determining unhealthy eating patterns from the preschool years onward.

Another key contribution of the current study, which has received less scientific attention, is the differentiation between types of screen-based sedentary behaviors and the examination of their specific mediating roles. TV viewing emerged as the most prevalent screen device, closely followed by smartphones and tablets, and weekend exposure substantially exceeded weekday exposure, highlighting unstructured leisure periods as particularly critical windows of risk. In the current study, screen-based behaviors partially mediated the association between maternal BMI and UPF consumption (15–90% of total effects), with especially strong indirect effects for snack intake (50–90%). These results suggest that media-related contexts constitute salient environments in which UPF is promoted, offered, or consumed, consistent with evidence that higher screen time across early and middle childhood is associated with less healthy dietary patterns and increased obesity risk [5,29]. The observed patterns reinforce the notion that screen time exposure runs as a behavioral pathway through which parental features contribute to obesogenic dietary profiles in young children.

Parental education also exhibited meaningful indirect pathways through screen-based behaviors, although with smaller magnitudes than those observed for BMI (9–23% indirect effects). Lower educational levels were associated with higher screen time and higher UPF consumption, whereas higher paternal education, in particular, appeared more protective against packaged and fast-food intake, as was previously pointed out. Despite of much of the literature has traditionally focused on mothers, recent studies highlight the specific contribution of fathers to young children’s eating habits [30]; for example, research with preschool-aged children shows that parental food literacy and feeding practices in the family context are central to the development of healthy eating patterns, as parents decide which foods are purchased, available at home, and routinely offered, including the extent to which ultra-processed products are part of everyday meals and snacks [25,26]. In this regard, higher paternal education is likely to be associated with better nutrition knowledge, greater awareness of the health risks of UPF, and stronger skills to interpret food labels and marketing, which may translate into more critical choices and lower availability of UPF in the household. Indeed, parental nutrition literacy and educational level are inversely associated with childhood obesity [28] and unhealthy dietary patterns, particularly in the context of food insecurity or socioeconomic vulnerability [7,31]. Moreover, the results converge with evidence that a more favorable home food environment and lower parental stress are linked to healthier diet patterns in families of preschool children [4,32], suggesting that education and literacy may shape not only food availability and quality, but also the regulation of children’s media use, the structuring of mealtime routines, and the balance between active and sedentary leisure. Although parental education and children’s sedentary behaviors together explained between 4% and 14% of the variance in UPF scores, this level of explained variance is typical of complex behavioral models in real-world family contexts, where various other factors also exert influence. Nevertheless, it underscores the importance of these pathways for early prevention initiatives.

Indeed, higher UPF consumption among children may reflect the combined influence of the school food environments, food marketing, daily routines, and household food practices as a result of familial adaptation to a new social and economic context where UPF is widely available, affordable, shelf-stable, and heavily marketed. Furthermore, these products often require minimal preparation and are readily accepted by children, making them practical choices given time constraints and structural barriers faced by households [28,31]. In the current study, after adjustment for parental educational level, the magnitude of several associations was reduced, indicating that SES partially explains differences in UPF consumption among children. Previous studies corroborated that lower parental education and income are often associated with higher UPF consumption among children [31], highlighting that parental education may influence nutrition knowledge, food purchasing behaviors, and awareness of dietary recommendations [6]. However, controversial research evidence from Southern European countries has also suggested that higher UPF intake may also occur among families with higher educational levels, particularly at younger ages [33]. For example, data of the DELICIOUS project revealed that almost all children and adolescents from five Mediterranean countries, including Portugal, consume unhealthy UPFs on a daily basis, with higher intakes among older adolescents, those with obesity, offspring of more highly educated parents and those characterized by more frequent out-of-home eating, greater snack consumption, higher screen time and an overall less healthy lifestyle profile [33,34]. The aforementioned results inconsistency and the relatively small magnitudes of associations observed in the present study emphasize the complexity of socioeconomic gradients in UPF consumption and suggest that SES alone does not fully account for those related differences.

It should also be underlined that dietary patterns are commonly influenced by food insecurity, which has been consistently affected by economic constraints, educational background, cultural factors, and changes in lifestyle [35,36]. For example, in Portugal, recent studies revealed that food insecurity has been linked to lower overall dietary quality and an increased dependence on energy-dense, nutrient-poor foods among children, which may, in turn, exacerbate household UPF consumption [36,37]. Therefore, from a public health perspective, findings of the current study support multilevel, family-centered interventions that could integrate nutrition, screen use, and physical activity within coherent lifestyle frameworks. For example, at the child and family levels, future strategies might include promoting regular family meals without screens, screen-free family meals; prioritizing minimally processed foods and water as default options; and reducing the availability and prominence of UPF at home. Such approaches are aligned with calls for a unified global public health response to the commercial determinants of UPF consumption and the need to counter corporate drivers of obesogenic diets [10], as well as evidence that regular family meals are associated with healthier dietary profiles and lower sedentary and unhealthy eating behaviors among school-aged children [38] and their parents/tutors [13,15]. Consistent with recommendations from the Portuguese Society of Pediatrics and the American Academy of Pediatrics [39], parents should be supported in establishing age-appropriate limits on screen time use, avoiding digital media, particularly during meals and before bedtime, and ensuring that screen exposure does not displace sleep, active play, or social interaction, mainly during weekends, when screen-based sedentary behaviors tend to achieve higher levels among pediatric people.

Recent combined research [28,33,40] indicates that higher UPF consumption is notably prevalent among younger populations and is closely linked with specific social patterns and unhealthy lifestyle profiles. For example, in the aforementioned systematic review of nationally representative surveys from thirty-two countries [28], studies demonstrated that higher UPF intake is consistently and independently associated with younger age, urban residence, non-married status, specific ethnic and regional groups, and, in a heterogeneous fashion across settings, with education, income, and food insecurity, thereby revealing marked sociodemographic inequalities in exposure to UPFs and related non-communicable disease. Interestingly, from the Portuguese geographic settings [6], the authors reported that UPFs contribute approximately one quarter of total daily energy intake, with particularly high intakes among youth and adults with higher educational attainment, and with yogurts containing additives, soft drinks, and processed meats emerging as the main dietary sources, differentially distributed according to age, sex, and education level. Thus, taken together and combined with findings of the current study, we suggest that UPF consumption is structurally embedded in contemporary dietary patterns—particularly among younger age groups—and underscore the need for comprehensive, multi-level public health strategies to promote the replacement of UPFs by minimally processed foods within traditional dietary patterns such as the Mediterranean diets among children. Attention is also due to the higher rates of sedentary habits, since research pointed out that school-aged children exhibiting a cluster of sedentary habits and irregular eating behaviors [13]—specifically characterized by prolonged screen exposure, digital distraction during meals, social isolation while eating, and a lack of adherence to structured meal patterns or domestic food preparation—demonstrating higher likelihood of ultra-processed consumption [41] as well as deficit in the intake of minimally processed foods.

### Strengths and Limitations

Despite several strengths, including a relatively large sample of preschool-aged children, the use of the original standardized tool to assess UPF consumption, and the robust mediating statistical models, the current study also has several limitations that have to be recognized. The cross-sectional design excludes causal inference, and dietary intake was assessed via parental report of consumption on the previous day, which may be affected by recall and social desirability biases and may not reflect usual intake. In addition, parental anthropometric data were self-reported rather than objectively measured, which may introduce measurement error, particularly social desirability bias, leading to either underestimation or overestimation of BMI levels. Moreover, although the sample size was relatively large, it may not be fully representative of all socioeconomic and cultural contexts, potentially restricting the generalizability of the above-noted findings. In the present study, participation was voluntary and based on convenience sampling, which could have implications for the interpretation of the findings. In fact, parents who declined to participate may systematically differ from those who agreed in several respects that are directly related to children’s UPF intake and sedentary behaviors, such as health awareness, time availability, trust in research, or interest in nutrition- and lifestyle-related topics. On the other hand, the sample is also diverse in terms of chronological age, which might limit the applicability of the aforementioned results since age may exert some influence on both ultra-processed food intake and sedentary behaviors among children, particularly during the transition from preschool to primary school. Of note, in the present study, chronological age was not the primary conceptual focus; rather, our interest was focused on the specific educational cycle, such as preschool, which is characterized by a peculiar and distinctive organizational and pedagogical structure, with highly standardized daily routines and school policies, as well as closely integrated family arrangements around care and feeding. Finally, securing accurate measurements of dietary intake across populations is challenging; thus, like all dietary methods, 24 h recalls are subject to random errors that lower the precision and systematic errors that can reduce accuracy at each stage of the measurement protocol, and, therefore, this has to be also recognized as a potential limitation of the present study. Therefore, future studies should apply the UPF instrument on multiple days of the week to better capture habitual intake and reduce day-to-day variability, as well as employ longitudinal designs, objective device-based measures of sedentary behaviors exposure and physical activity, and repeated, detailed dietary assessments are warranted to clarify temporal dynamics and to examine whether reductions in screen time and UPF availability effectively attenuate the intergenerational transmission of obesogenic dietary patterns.

## 5. Conclusions

In this study, screen-based sedentary behaviors partially mediated the pathways between maternal BMI and children’s ultra-processed food (UPF) intake, with the strongest mediation observed for snack consumption. Lower parental education and higher parental BMI were associated with greater screen use and higher UPF intake, with screen time contributing to the indirect effects of parental education, while parental education and children’s sedentary behaviors together just explained a modest proportion of the variance in the UPF scores. These findings support implementing family-centered strategies that jointly reduce preschoolers’ screen time and UPF exposure—especially at the weekends—and suggest prioritizing families with higher BMI and lower education in early obesity-prevention interventions, mitigating early obesity trajectories.

## Figures and Tables

**Table 1:** Descriptive statistics of screen time-related sedentary behaviors and ultra-processed food consumption, and associations with parental BMI and educational level.

Variables	Observed Range (Min–Max)	M ± SD	Mothers’ BMI	Mothers’ Years of Schooling	Fathers’ BMI	Father’s Years of Schooling
**Television (min/day)**						
Weekdays	0–250	51.73 ± 40.27	0.08 *	−0.08 *	0.01	−0.09 *
Weekend days	0–250	97.48 ± 54.97	0.11 **	−0.05	0.00	−0.05
**Gaming consoles (min/day)**						
Weekdays	0–90	0.82 ± 5.45	0.07 *	−0.03	0.02	−0.04
Weekend days	0–150	5.48 ± 19.54	0.04	−0.09 **	0.02	−0.03
**Computers (min/day)**						
Weekdays	0–90	0.85 ± 6.51	−0.02	−0.01	−0.02	−0.02
Weekend days	0–150	3.72 ± 13.63	0.06	−0.04	−0.01	0.01
**Tablets (min/day)**						
Weekdays	0–250	13.26 ± 28.86	0.10 **	−0.14 **	0.08 *	−0.05
Weekend days	0–250	24.89 ± 42.98	0.10 **	−0.11 **	0.09 *	−0.06
**Smartphones (min/day)**						
Weekdays	0–250	20.39 ± 30.97	0.08 *	−0.14 **	0.09 *	−0.23 **
Weekend days	0–250	34.42 ± 42.61	0.11 **	−0.16 **	0.09 *	−0.26 **
**UPF drinks and yogurts**						
Previous 24 h	0–6	1.15 ± 1.14	0.15 **	−0.28 **	0.09 **	−0.25 **
Outside the home	0–6	0.31 ± 0.70	0.08 *	−0.07 *	0.02	−0.11 **
During screen time	0–2	0.08 ± 0.30	0.12 **	−0.06	0.01	−0.08 *
**UPF packaged and fast foods**						
Previous 24 h	0–11	1.05 ± 1.43	0.14 **	−0.14 *	0.04	−0.19 **
Outside the home	0–9	0.22 ± 0.76	0.03	−0.04	−0.02	−0.09 **
During screen time	0–6	0.06 ± 0.37	0.05	−0.03	0.04	−0.04
**UPF sweet and salty snacks**						
Previous 24 h	0–10	1.42 ± 1.33	0.03	−0.06	0.02	−0.13 **
Outside the home	0–10	0.18 ± 0.68	0.04	−0.02	−0.01	−0.05
During screen time	0–3	0.12 ± 0.40	0.04	−0.05	−0.01	−0.12 *
**UPF total score**						
Previous 24 h	0–27	3.61 ± 2.87	0.15 **	−0.21 **	0.06	−0.25 **
Outside the home	0–20	0.70 ± 1.50	0.07 *	−0.04	−0.01	−0.11 **
During screen time	0–7	0.26 ± 0.75	0.09 **	−0.06	0.02	−0.11 **

Note: * *p* < 0.05, ** *p* < 0.01

**Table 2:** Mediation analysis of weekdays screen-related time on the association between parents’ body mass index and children’s ultra-processed food intake.

Parameter	Standardized Estimate	Standard Error	95% CI (Lower, Upper)
**Direct effects**			
Mother’s BMI → UPF DY	0.115	0.044	0.033, 0.202 *
Father’s BMI → UPF DY	0.036	0.038	−0.038, 0.115
Mother’s BMI → UPF PFF	0.128	0.051	0.030, 0.232 *
Father’s BMI → UPF PFF	−0.014	0.035	−0.083, 0.053
Mother’s BMI → UPF SSS	0.021	0.052	−0.074, 0.134
Father’s BMI → UPF SSS	−0.006	0.034	−0.072, 0.055
**Total indirect effects**			
Mother’s BMI → SSB → UPF DY	0.030	0.012	0.009, 0.059 *
Father’s BMI → SSB → UPF DY	0.017	0.010	−0.001, 0.040
Mother’s BMI → SSB → UPF PFF	0.023	0.010	0.007, 0.047 *
Father’s BMI → SSB → UPF PFF	0.009	0.008	−0.006, 0.027
Mother’s BMI → SSB → UPF SSS	0.021	0.010	0.004, 0.040 *
Father’s BMI → SSB → UPF SSS	0.009	0.008	−0.003, 0.029
**Total effects**			
Mother’s BMI → UPF DY	0.145	0.043	0.061, 0.232 *
Father’s BMI → UPF DY	0.054	0.038	−0.021, 0.130
Mother’s BMI → UPF PFF	0.151	0.050	0.053, 0.250 *
Father’s BMI → UPF PFF	−0.005	0.035	−0.073, 0.065
Mother’s BMI → UPF SSS	0.042	0.053	−0.054, 0.152
Father’s BMI → UPF SSS	0.003	0.034	−0.064, 0.067

Note: BMI = body mass index; SSB = screen-related sedentary behavior; UPF = ultra-processed food intake; DY = drinks and yogurts; PFF = packaged and fast foods; SSS = sweet and salty snacks; SE = standard error; * effect is significant at *p* < 0.05, when zero is not included in 95% confidence interval.

**Table 3:** Mediation analysis of weekend screen-related time on the association between parents’ body mass index and children’s ultra-processed food intake.

Parameter	Standardized Estimate	Standard Error	95% CI (Lower, Upper)
**Direct effects**			
Mother’s BMI → UPF DY	0.109	0.043	0.027, 0.194 *
Father’s BMI → UPF DY	0.039	0.038	−0.034, 0.114
Mother’s BMI → UPF PFF	0.122	0.050	0.033, 0.228 *
Father’s BMI → UPF PFF	−0.016	0.034	−0.085, 0.051
Mother’s BMI → UPF SSS	0.004	0.050	−0.084, 0.107
Father’s BMI → UPF SSS	−0.008	0.033	−0.074, 0.057
**Total indirect effects**			
Mother’s BMI → SSB → UPF DY	0.034	0.012	0.012, 0.059 *
Father’s BMI → SSB → UPF DY	0.017	0.010	−0.008, 0.038
Mother’s BMI → SSB → UPF PFF	0.028	0.009	0.011, 0.047 *
Father’s BMI → SSB → UPF PFF	0.010	0.008	−0.004, 0.028
Mother’s BMI → SSB → UPF SSS	0.037	0.011	0.018, 0.064 *
Father’s BMI → SSB → UPF SSS	0.009	0.009	−0.009, 0.028
**Total effects**			
Mother’s BMI → UPF DY	0.143	0.043	0.059, 0.228 *
Father’s BMI → UPF DY	0.055	0.038	−0.008, 0.038
Mother’s BMI → UPF PFF	0.150	0.050	0.011, 0.047 *
Father’s BMI → UPF PFF	−0.007	0.035	−0.004, 0.028
Mother’s BMI → UPF SSS	0.041	0.052	0.018, 0.064 *
Father’s BMI → UPF SSS	0.001	0.034	−0.009, 0.028

Note: BMI = body mass index; SSB = screen-related sedentary behavior; UPF = ultra-processed food intake; DYs = drinks and yogurts; PFFs = packaged and fast foods; SSSs = sweet and salty snacks; SE = standard error; * effect is significant at *p* < 0.05, when zero is not included in 95% confidence interval.

**Table 4:** Mediation analysis of weekdays screen-related time on the association between parents’ educational level and children’s ultra-processed food intake.

Parameter	Standardized Estimate	Standard Error	95% CI (Lower, Upper)
**Direct effects**			
Mother’s EL → UPF DY	−0.193	0.041	−0.275, −0.112 *
Father’s EL → UPF DY	−0.114	0.037	−0.188, −0.040 *
Mother’s EL → UPF PFF	−0.060	0.039	−0.138, 0.011
Father’s EL → UPF PFF	−0.139	0.041	−0.224, −0.061 *
Mother’s EL → UPF SSS	0.002	0.040	−0.078, 0.075
Father’s EL → UPF SSS	−0.092	0.046	−0.194, −0.014 *
**Total indirect effects**			
Mother’s EL → SSB → UPF DY	−0.021	0.010	−0.044, −0.003 *
Father’s EL → SSB → UPF DY	−0.034	0.012	−0.059, −0.011 *
Mother’s EL → SSB → UPF PFF	−0.015	0.009	−0.036, 0.001
Father’s EL → SSB → UPF PFF	−0.022	0.011	−0.047, −0.002 *
Mother’s EL → SSB → UPF SSS	−0.019	0.010	−0.040, −0.002 *
Father’s EL → SSB → UPF SSS	−0.023	0.012	−0.050, −0.001 *
**Total effects**			
Mother’s EL → UPF DY	−0.214	0.040	−0.294, −0.137 *
Father’s EL → UPF DY	−0.148	0.037	−0.221, −0.078 *
Mother’s EL → UPF PFF	−0.075	0.038	−0.155, −0.007 *
Father’s EL → UPF PFF	−0.161	0.038	−0.237, −0.089 *
Mother’s EL → UPF SSS	−0.016	0.039	−0.098, 0.059
Father’s EL → UPF SSS	−0.114	0.042	−0.205, −0.042 *

Note: EL = educational level; SSB = screen-related sedentary behavior; UPF = ultra-processed food intake; DYs = drinks and yogurts; PFFs = packaged and fast foods; SSSs = sweet and salty snacks; SE = standard error; * effect is significant at *p* < 0.05, when zero is not included in 95% confidence interval.

**Table 5:** Mediation analysis of weekend screen-related time on the association between parents’ educational level and children’s ultra-processed food intake.

Parameter	Standardized Estimate	Standard Error	95% CI (Lower, Upper)
**Direct effects**			
Mother’s EL → UPF DY	−0.195	0.040	−0.271, −0.114 *
Father’s EL → UPF DY	−0.117	0.036	−0.190, −0.050 *
Mother’s EL → UPF PFF	−0.058	0.040	−0.133, 0.021
Father’s EL → UPF PFF	−0.137	0.040	−0.281, −0.061 *
Mother’s EL → UPF SSS	0.006	0.041	−0.075, 0.085
Father’s EL → UPF SSS	−0.086	0.043	−0.168, −0.002 *
**Total indirect effects**			
Mother’s EL → SSB → UPF DY	−0.019	0.010	−0.041, −0.002 *
Father’s EL → SSB → UPF DY	−0.031	0.014	−0.060, −0.007 *
Mother’s EL → SSB → UPF PFF	−0.016	0.009	−0.036, −0.002 *
Father’s EL → SSB → UPF PFF	−0.024	0.013	−0.053, −0.001 *
Mother’s EL → SSB → UPF SSS	−0.023	0.010	−0.047, −0.006 *
Father’s EL → SSB → UPF SSS	−0.024	0.013	−0.052, −0.001 *
**Total effects**			
Mother’s EL → UPF DY	−0.214	0.040	−0.291, −0.135 *
Father’s EL → UPF DY	−0.148	0.036	−0.219, −0.081 *
Mother’s EL → UPF PFF	−0.074	0.039	−0.151, 0.004
Father’s EL → UPF PFF	−0.161	0.038	−0.237, −0.090 *
Mother’s EL → UPF SSS	−0.017	0.040	−0.097, 0.061
Father’s EL → UPF SSS	−0.111	0.042	−0.190, −0.027

Note: EL = educational level; SSB = screen-related sedentary behavior; UPF = ultra-processed food intake; DY = drinks and yogurts; PFF = packaged and fast foods; SSS = sweet and salty snacks; SE = standard error; * effect is significant at *p* < 0.05, when zero is not included in 95% confidence interval.

## Data Availability

There are restrictions on the availability of data for this trial, due to the signed consent agreements around data sharing, which only allow access to external researchers for studies following the project’s purposes. Requestors wishing to access the trial data used in this study can make a request to drdc@uc.pt.
